# Electrochemical behavior of dye-linked L-proline dehydrogenase on glassy carbon electrodes modified by multi-walled carbon nanotubes

**DOI:** 10.3762/bjnano.1.16

**Published:** 2010-12-14

**Authors:** Haitao Zheng, Leyi Lin, Yosuke Okezaki, Ryushi Kawakami, Haruhiko Sakuraba, Toshihisa Ohshima, Keiichi Takagi, Shin-ichiro Suye

**Affiliations:** 1 School of Environmental Science and Chemical Engineering, Tianjin Polytechnic University, Tianjin 300160, P.R. China; 2 Department of Applied Chemistry and Biotechnology, Graduate School of Engineering, University of Fukui, Fukui 910-8507, Japan; 3 Analytical Research Center for Experimental Sciences, Saga University, Saga 840-8502, Japan; 4 Department of Applied Biological Science, Faculty of Agriculture, The University of Kagawa, Kagawa 761-0795, Japan; 5 Microbial Genetics Division, Institute of Genetic Resources, Faculty of Agriculture, Kyushu University, Fukuoka 812-8581, Japan; 6 Wakasa Wan Energy Research Center, Tsuruga 914-0192, Japan

**Keywords:** dye-linked L-proline dehydrogenase, electrocatalysis, electron transfer, multi-walled carbon nanotube

## Abstract

A glassy carbon electrode (GC) was modified by multi-walled carbon nanotubes (MWCNTs). The modified electrode showed a pair of redox peaks that resulted from the oxygen-containing functional groups on the nanotube surface. A recombinant thermostable dye-linked L-proline dehydrogenase (L-proDH) from hyperthermophilic archaeon (*Thermococcus profundus*) was further immobilized by physical adsorption. The modified electrode (GC/MWCNTs/L-proDH) exhibited an electrocatalytic signal for L-proline compared to bare GC, GC/L-proDH and GC/MWCNTs electrodes, which suggested that the presence of MWCNTs efficiently enhances electron transfer between the active site of enzyme and electrode surface. The immobilized L-proDH showed a typical Michaelis–Menten catalytic response with lower apparent constant.

## Introduction

As an essential amino acid for the proper functioning of tendons and joints in the human body, the quick and sensitive determination of L-proline is of importance for both pharmaceutical and food industries [1]. Classical fluorescence [2] and chromatography methods [3–4] can determine the substrate on a relatively sensitive scale, but the pre-treatment procedures are tedious and these two methods always require considerable time for analysis.

In recent years, researchers have paid much more attention to the construction of electrochemical enzyme biosensors for the analysis of amino acids [5–7], and several electrochemical biosensing systems for L-proline and D-proline determination have been reported [8–9]. In the construction of enzyme biosensors, efficient communication between the active site of enzyme molecule and the electrode surface is one of the most important factors for sensor performance. As the protein shell always prevents the direct electron transfer from the active site of the enzyme, specific immobilization strategies, including electron mediators, should be considered [10–12].

In the last decade, the use of nano materials, especially carbon nanotubes (CNTs), in the construction of enzyme biosensors has received considerable attention. Because of their excellent mechanical and electrochemical properties [13–14], CNTs can mediate the electron transfer between an electrode and a number of electroactive substances such as hydrogen peroxide, ascorbic acid and dopamine, and accelerate surface electrochemical reactions [15]. Direct electron transfer between the active site of several biomacromolecules and the electrode surface can also be set up with the aid of CNTs [16–17]. Because the application of CNTs can dramatically improve the sensitivity of electrochemical sensors, more and more analytical chemists have focused their attention on CNTs-modified electrodes for the detection of various types of chemical or biochemical substances [18–23]. Some amino acid biosensors have already been reported based on CNTs-modified electrodes [24–27].

In our previous work, the recombinant thermostable dye-linked L-proline dehydrogenase (L-proDH) from hyperthermophilic archaeon (*Thermococcus profundus*) was used to fabricate L-proline sensor based electrodes modified by layer-by-layer self-assembly immobilization of dye-linked L-proDH and ferrocene-labeled redox polyelectrolytes [9,28], which exhibited an amperometric current response to L-proline. Because electroactive ferrocene groups were used as the electron mediator, the above sensors should operate at an applied potential of +0.45 V to satisfy the redox reaction of ferrocene. In order to improve the sensitivity of electrochemical L-proline biosensor at lower applied potentials, multi-walled carbon nanotubes (MWCNTs) were employed in this work. Both MWCNTs and L-proDH were immobilized on the electrode surface, and the electrochemical behavior of the dye-linked L-proDH on the electrode modified by MWCNTs was investigated.

## Results and Discussion

### Morphology of MWCNT–modified GC electrode surface

**Figure 1:**
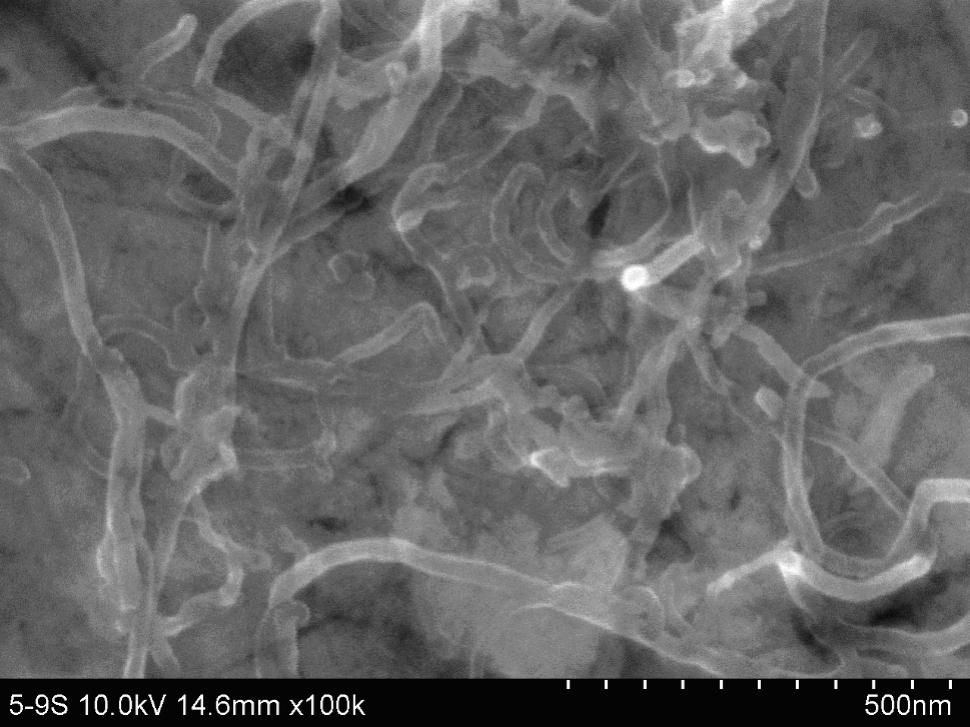
SEM image of MWCNTs-modified GC electrode.

The SEM image of MWCNTs on a GC electrode surface is shown in Figure 1. Nanotubes of 10−20 nm diameter were observed. Some nanotubes were twisted together, and became indiscernible. The length of the nanotubes used in this work was 5−15 μm, the shorter nanotubes observed can be attributed to the long-time ultrasonication (30 min) used.

### Electrochemical behavior of L-proDH on MWCNTs–modified GC electrode

**Figure 2:**
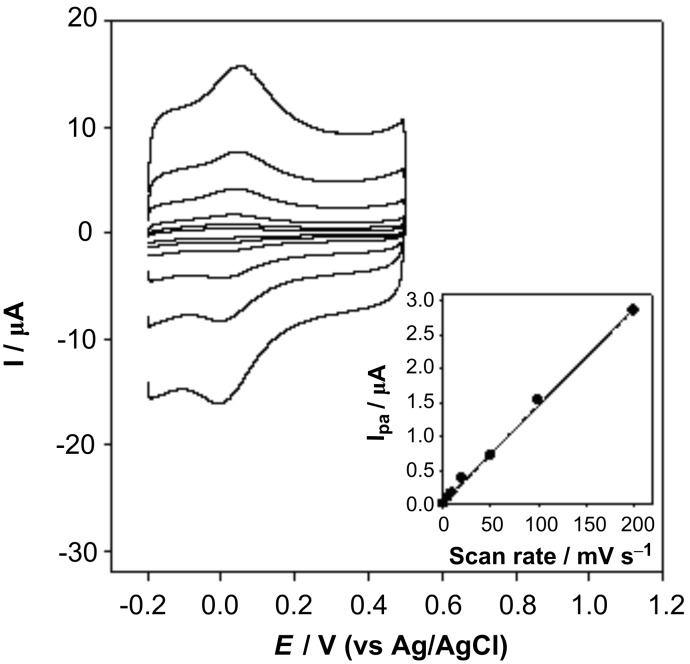
Cyclic voltammograms of the GC/MWCNTs electrode in phosphate buffer at different scan rates (5, 10, 20, 50, 100, 200 mV s^−1^ from inner to outer). Insert: The relationship between I_pa_ and scan rate.

The electrochemical properties of GC/MWCNTs were first investigated by cyclic voltammetry with K_3_Fe(CN)_6_ as the probe. Typical reversible cyclic voltammograms were observed for both the bare and MWCNTs modified electrodes (data not shown here). It was clearly demonstrated that the current obtained on the GC/MWCNTs was nearly 2.5 times greater than that with a bare GC. The larger current response indicated an increased electrode surface or a fast electron process after modification by MWCNTs. The cyclic voltammograms of the GC/MWCNTs electrode in phosphate buffer (0.3 M, pH 7.5) at different scan rates (from 5 to 200 mV s^−1^) are displayed in Figure 2. There was a pair of redox peaks at a formal potential (*E*^0’^=(*E*_pa_+*E*_pc_)/2) of approximately +0.02 V. In the insert figure of Figure 2, the anodic peak current increases linearly with the scan rate, which indicates a diffusion-free electrochemical process. Because the bare GC electrode did not show any redox signal under the same experimental conditions, the redox peaks obtained on GC/MWCNTs can be attributed to the oxidation/reduction of some functional groups on the surface of MWCNTs.

**Figure 3:**
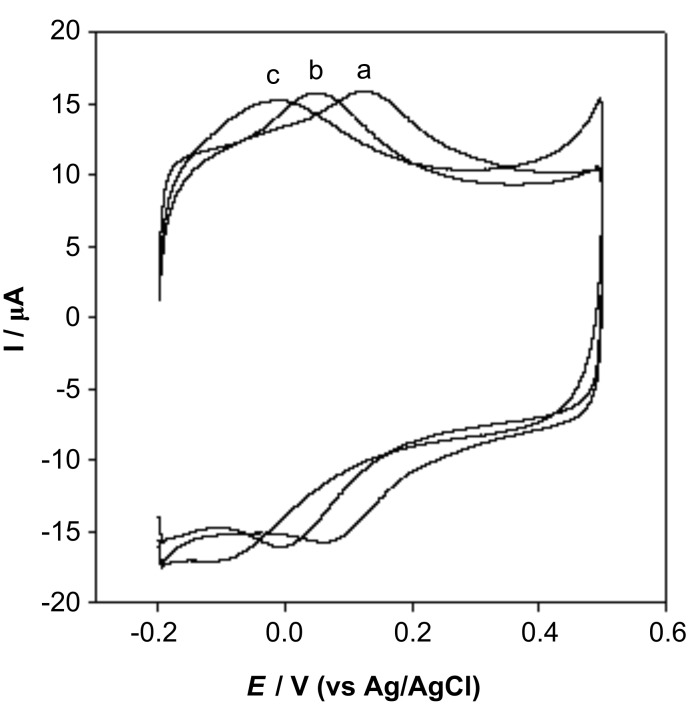
Cyclic voltammograms of the GC/MWCNTs electrode at pH 6.0 (a), pH 7.5 (b) and pH 9.0 (c) phosphate buffer at a scan rate of 200 mV s^−1^.

The cyclic voltammograms of the GC/MWCNTs electrode in phosphate buffer at different pHs were also studied (Figure 3). The redox peak potentials were dependent on the buffer pH, and a linear relationship between the formal potential *E*^0’^ and buffer pH was found, with the linear regression equation of *E*^0’^(V) = 0.4001−0.0508 pH (correlation coefficient, r = 0.9984). Similar results were found by other groups [29–30] and indicate that an equal number of protons and electrons are involved in the redox reaction. This is ascribed to the oxidation/reduction process by some oxygen-containing functional groups on nanotube surface [31].

**Figure 4:**
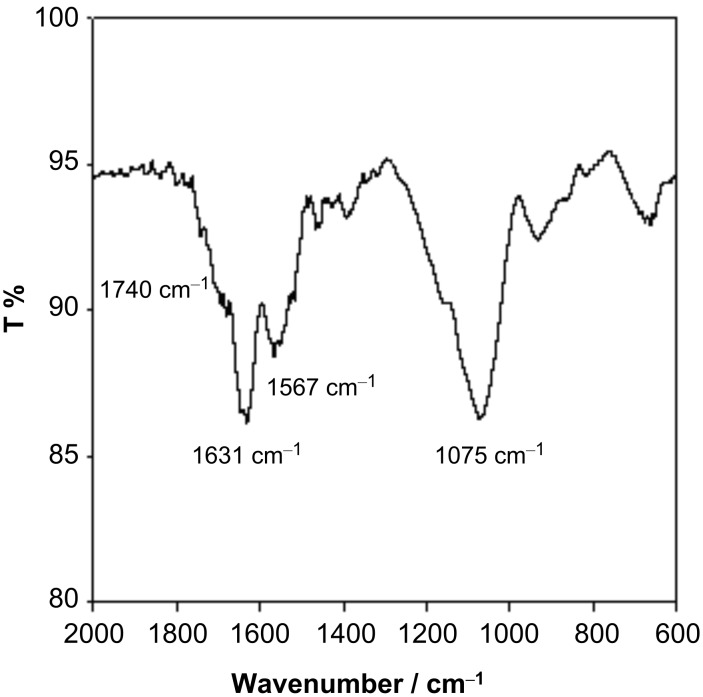
IR spectra of MWCNTs sample.

In the IR spectrum of the MWCNTs sample shown in Figure 4, the adsorption peaks at 1740 cm^−1^ and 1075 cm^−1^ are due to a carbonyl group and C–O bond, respectively. This also indicates the existence of oxygen-containing groups on nanotube surface. As commercial MWCNTs were used in this research, some oxygen-containing groups must have been formed after preparation. Zhang et al. also found these kinds of functional groups when freshly prepared nanotubes were exposed to air after one day, and pointed out that the redox waves of these groups cannot be eliminated by removal of the dissolved oxygen in buffer [31].

**Figure 5:**
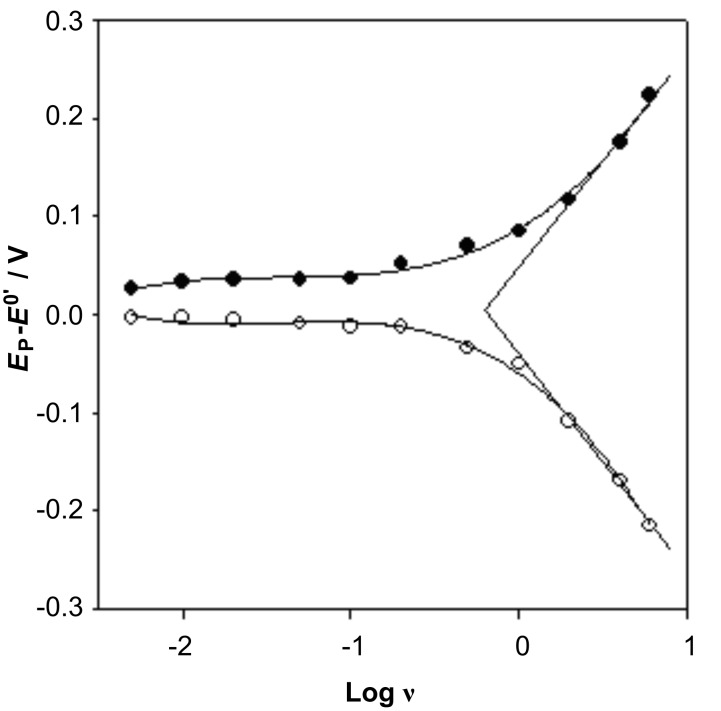
Plots of anodic peak potential and cathodic peak potential against the logarithm of scan rate. All experiments were carried out on GC/MWCNTs in phosphate buffer (pH 7.5).

The influence of scan rate on the anodic and cathodic peak positions is shown in Figure 5. The electron transfer parameter (α) of the oxygen-containing functional groups was calculated to be 0.5, and the number of electrons (n) was estimated to be 0.61, according to Laviron’s model [32], which means that about one electron was involved in the redox process. This is consistent with the results by Barisci et al. [29], who assigned the redox process on the nanotube surface to the following reaction, =C=O + H^+^ + e^−^ = =COH.

### Electrochemical behavior of L-proDH on MWCNTs-modified GC electrode

**Figure 6:**
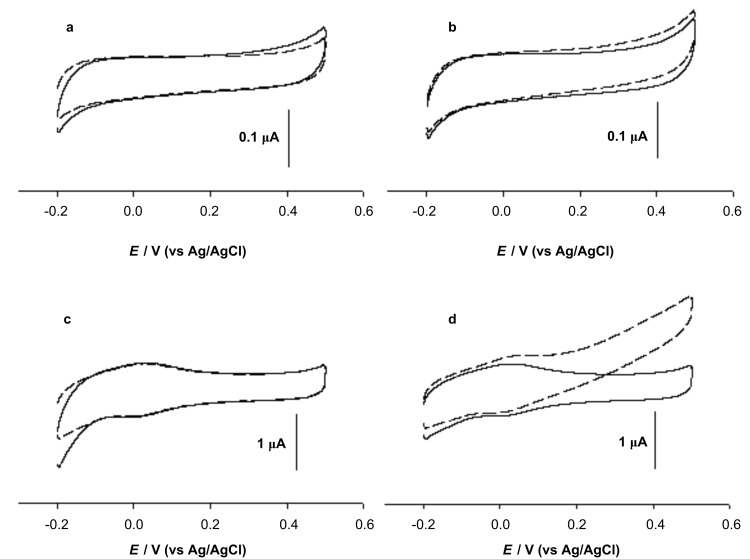
Cyclic voltammograms of GC (a), GC/L-proDH (b), GC/MWCNTs (c) and GC/MWCNTs/L-proDH (d) electrodes in phosphate buffer in the absence (solid line) and presence (dash line) of 10 mM L-proline at a scan rate of 5 mV s^−1^.

Dye-linked L-proDH was further immobilized on a MWCNTs-modified electrode, and its electrochemical behavior was studied. Typical cyclic voltammograms of GC, GC/L-proDH, GC/MWCNTs and GC/MWCNTs/L-proDH electrodes in the absence and the presence of L-proline are shown in Figure 6. No catalytic current response was observed with the GC, GC/L-proDH and GC/MWCNTs electrodes in the potential region of −0.2 to +0.5 V in the presence of substrate (Figure 6a, Figure 6b and Figure 6c). Although the GC/MWCNTs electrode showed a pair of redox peaks due to the oxygen-containing functional groups on the nanotube surface, these functional groups cannot catalyze the oxidation of L-proline by themselves. In Figure 6d, the GC/MWCNTs/L-proDH electrode exhibited an apparently catalytic response to the substrate, and the anodic current increased compared with the cyclic voltammograms on other electrodes shown in Figure 6a, Figure 6b and Figure 6c. In the case of the GC/MWCNTs/L-proDH electrode, L-proline was first oxidized at the FAD sites of the immobilized enzyme molecules, and intramolecular electron transfer took place between the FAD sites of L-proDH and the electrode with the help of the oxygen-containing functional groups on the nanotube surface. This suggests that the electrical communication between the active sites of enzyme and electrode was set up with the help of the nanotubes.

**Figure 7:**
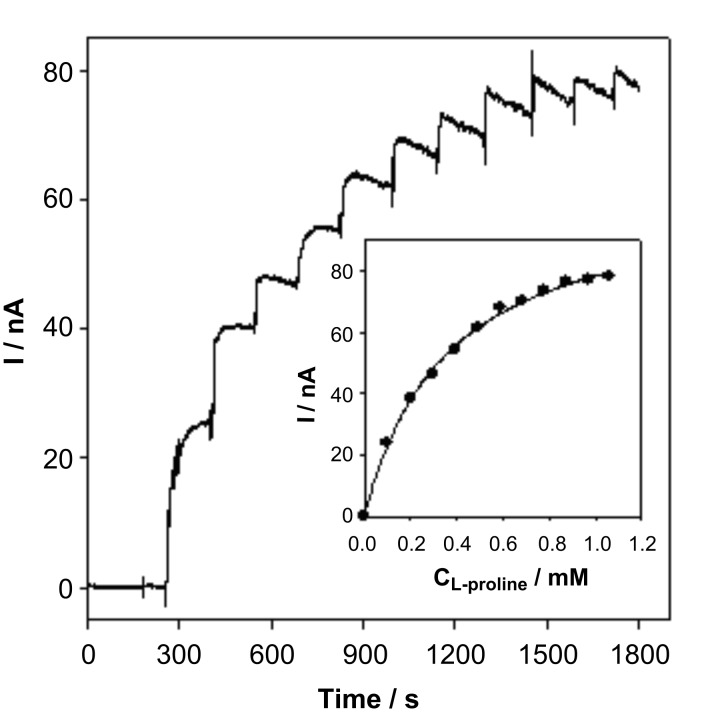
Amperometric response of GC/MWCNTs/L-proDH to the successive addition of 0.1 mM L-proline at an applied potential of +0.2 V. Insert: The relationship between the concentration of L-proline and catalytic current response.

The amperometric response of the GC/MWCNTs/L-proDH electrode to L-proline was further investigated at an applied potential of +0.2 V. The current response increased with successive additions of substrate and with stirring as shown in Figure 7, and the response occurred within 15 s. The relationship between C_L-proline_ and I is displayed in the insert figure of Figure 7, and the current response appears to be saturated when the substrate concentration was larger than 1 mM as a result of the Michaelis–Menten kinetic mechanism. Also shown in Figure 7, the amperometric signal seemed to be unstable after the first few injections. This may be a result of the larger injection interference when the enzymatic reaction was closed to saturation.

The amperometric response was about 0.87 μA cm^−2^ for 1 mM L-proline with a detection limit of 0.05 mM (S/N = 3), which was much higher than that obtained in previous work which used a ferrocene-labeled redox polymer as the electron mediator [9,28], and the applied potential was also considerably decreased (from +0.45 V to +0.2 V). This can be attributed to the presence of the oxygen-containing functional groups on MWCNTs, which rapidly shuttled the electron between the active sites of L-proDH and electrode surface.

**Figure 8:**
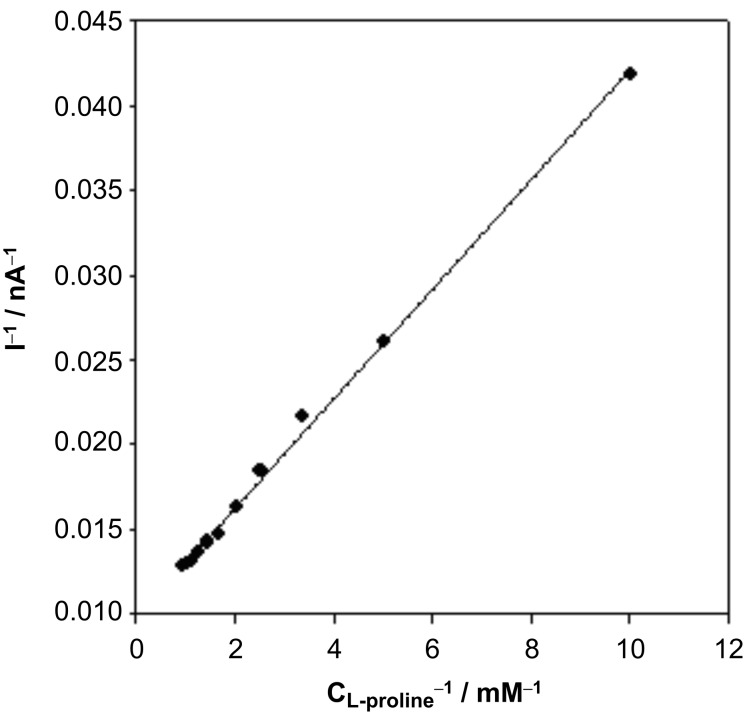
Lineweaver–Burke plot of 1/I vs 1/C_L-proline_.

The Lineweaver–Burk curve of 1/I vs 1/C_L-proline_ is plotted in Figure 8, and a linear relationship between 1/I vs 1/C_L-proline_ was clearly found, which is consistent with the following Lineweaver–Burk equation.

[1]
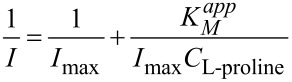


According to the slope and intercept of Lineweaver–Burk equation, the apparent Michaelis–Menten constant (K*_M_^app^*) of the immobilized L-proDH on MWCNTs-modified electrode was calculated to be 0.33 mM, which was lower than that of free L-proDH (2.05 mM) in buffer with DCPIP as the electron acceptor [33]. This indicated that fast electron transfer occurred between the oxygen-containing functional groups and the FAD active site of L-proDH.

## Conclusion

In this work, both MWCNTs and dye-linked L-proDH were immobilized on an electrode surface, and the electrochemical behavior of L-proDH was investigated. On the MWCNTs-modified electrode, a pair of broad redox peaks was observed, which were shifted to more positive values with decreasing pH. These peaks were probably due to the oxygen-containing functional groups on nanotube surface. Based on these oxygen-containing functional groups, electrical communication between the FAD active sites of L-proDH and electrode were set up, and the electrode immobilized with both MWCNTs and L-proDH exhibited a catalytic current response to L-proline at a relatively lower potential. The GC/MWCNTs/L-proDH electrode showed a typical Michaelis–Menten response to the addition of L-proline in amperometric measurements, and the apparent Michaelis–Menten constant (K*_M_^app^*) of the immobilized L-proDH on MWCNTs-modified electrode was lower than that of the free enzyme in buffer solution with DCPIP as the electron acceptor.

## Experimental

### Materials

Multi-walled carbon nanotubes (MWCNTs) with a diameter of 10–20 nm and length of 5–15 μm were purchased from Tokyo Chemical In. (Tokyo, Japan) and used as received. L-Proline and 2,6-dichlorophenolindophenol (DCPIP) were obtained from Wako Chemical In. (Tokyo, Japan), and glutaraldehyde (25% w/w solution for electron microscopy grade was obtained form Nacarai Chemical Co., (Kyoto, Japan). All the other chemicals were of analytical grade.

### Apparatus

An FT-IR spectrometer (JASCO-4100A, Tokyo, Japan) was used to characterize the MWCNTs sample. The IR pellet was made by mixing a small amount powdered KBr and the nanotube sample, and the IR spectra was recorded. A field emission scanning electron microscopy (SEM, Hitachi S-4800HS, Tokyo, Japan) was used to observe the surface of MWCNTs-modified electrode. All electrochemical experiments were performed on a potentiostat (CHI-800B, Austin, USA) connected to a personal computer. A typical three-electrode system was used, with a 3 mm diameter of glassy carbon electrode (GC) as the working electrode, a platinum wire as the counter electrode and Ag/AgCl (3 M NaCl) as the reference electrode. The GC electrodes were finely polished by 1.0 μm and 0.05 μm alumina, and cleaned by ultrasonication in Milli-Q water for 30 s before modification.

### Enzyme assay

Recombinant dye-linked L-proDH, from hyperthermophilic archaeon, was prepared and purified according to the literature method [33]. The enzyme has an FAD active site which catalyzes the oxidation of L-proline in the presence of an artificial electron acceptor. The activity of the enzyme was routinely determined spectrophotometrically by measuring the reduction rate of DCPIP at 595 nm in accord with the previously described procedures [33–34]. The reaction mixture was composed of 0.1 mM DCPIP, 100 mM L-proline and enzyme in Tris-HCl buffer (0.3 M, pH 7.5) with a total volume of 3.0 ml. The molar absorption coefficient of 2.15 × 10^4^ M^−1^ cm^−1^ was used to calculate the reduction of DCPIP, and one unit of activity was defined as the amount of enzyme that reduced one μmol of DCPIP per min at 50 °C. The protein concentration of the L-proDH solution was found to be 1.6 mg mL^−1^ based on the classical Bradford method, and the specific activity of enzyme was calculated to be 1.8 units mg^−1^.

### Preparation of MWCNTs and L-proDH modified electrodes

A MWCNTs solution was prepared by dispersing 1.0 mg of MWCNTs in 1.0 mL ethanol followed by ultrasonication for 30 min. A 10 μL aliquot of the MWCNTs dispersion solution was dropped onto the top of the pre-treated GC electrode, which was dried at room temperature for 2 h and is referred to as GC/MWCNTs.

L-proDH was immobilized on MWCNTs-modified GC electrode by physical adsorption. A 10 μL L-proDH solution was dropped onto a MWCNTs-modified electrode, and dried in a N_2_ stream at room temperature. The electrode with the immobilized enzyme was washed three times with Milli-Q water, and stored in phosphate buffer (10 mM, pH 7.2) at 4 °C when not in use. The electrode modified with both MWCNTs and L-proDH is referred to as GC/MWCNTs/L-proDH. In controlled experiments, L-proDH was immobilized on a bare GC electrode surface by a cross-linking method using glutaraldehyde as the linking agent [35–36]. A 300 μL L-proDH solution and 200 μL glutaraldehyde solution (0.5%, w/w) were mixed. A 12 μL of the above mixture was coated onto the bare GC electrode and dried in a N_2_ stream at room temperature. The electrode so obtained is referred to as GC/L-proDH.

### Electrochemical measurements

All electrochemical measurements were carried out in 0.3 M phosphate buffer (pH 7.5) at room temperature (20 °C). The buffer solution was purged with high-purity N_2_ for 15 min before measurements to eliminate the influence of dissolved oxygen. The amperometric detection was performed at +0.2 V vs Ag/AgCl, and the substrate was added after the current reached the baseline.
